# A Survey of Veterinarian Attitudes Toward Prepubertal Desexing of Dogs and Cats in the Australian Capital Territory

**DOI:** 10.3389/fvets.2019.00272

**Published:** 2019-08-21

**Authors:** Bronwyn Orr, Bidda Jones

**Affiliations:** ^1^Faculty of Science, Sydney School of Veterinary Science, The University of Sydney, Sydney, NSW, Australia; ^2^The Royal Society for the Prevention of Cruelty to Animals, Canberra, ACT, Australia

**Keywords:** Australia, cats, desexing, dogs, mandatory, prepubertal, veterinarians

## Abstract

Prepubertal desexing (neutering) has been a legal requirement for all cats and dogs in the Australian Capital Territory since 2001. All cats have to be desexed by 3 months of age, and all dogs are required to be desexed by 6 months of age. The role of veterinary attitudes and behaviors in the implementation of mandatory prepubertal desexing in the Australian Capital Territory is explored in this paper. An online survey was sent to all veterinarians registered in the Australian Capital Territory via the Veterinary Surgeons Board (VSB). The survey was designed as a cross-sectional study, hosted using the software REDcap^®^ and analyzed using statistical program R^®^. A response rate of 14.9% (52/350) of registered veterinarians was achieved. Only 10% of respondents (5/52) recommended that clients have their cat desexed at 3 months of age, the legal maximum age for desexing in the jurisdiction. However, 40% of veterinarians (21/52) thought prepubertal desexing was an appropriate management strategy of cats to prevent overpopulation. Just over one-third of all veterinarians who responded (18/52; 35%) were not aware that prepubertal desexing was mandatory in the Australian Capital Territory. We conclude that prepubertal desexing might be poorly supported by veterinarians in the Australian Capital Territory, even though pets are legally required to undergo prepubertal desexing. As a result, veterinarians may unintentionally be limiting access to this procedure. This has wider policy consequences for Australian and overseas jurisdictions which are considering introducing mandatory prepubertal desexing.

## Introduction

The practice of prepubertal desexing (PD) and early age desexing (EAD) of dogs and cats has been utilized in animal shelters since the 1980s as a tool to combat overpopulation (1). Although often used interchangeably, the two terms differ in intent. PD is primarily concerned with desexing animals before they reach sexual maturity or puberty, which is defined here as before 4 months of age in cats and 7 months in dogs (2). EAD on the other hand, is primarily concerned with desexing animals as early as is safely possible. In cats, this can be from 6 weeks of age or 800 g bodyweight for males and 1,000 g bodyweight for females. Dogs are also desexed from 6 weeks of age in EAD, but most practitioners wait until 1,000 g bodyweight is reached (3). The age and bodyweight used to determine time of desexing in EAD is based on clinician surgical and anesthetic experience (4).

The “traditional” age of desexing in cats and dogs is between 5 and 9 months of age (1, 5). A recent study into the attitudes and behaviors of Australian veterinarians toward PD reported that most veterinarians recommended desexing by 6 months of age (6). For most dogs desexed in this period, it will be prepubertal. However, many cats would have reached sexual maturity at this age, meaning a female cat could become pregnant prior to desexing. This eliminates one of the main benefits of desexing: avoiding unwanted pregnancies (7). Proponents of traditional desexing argue that it provides a safer anesthetic, reduced long-term medical or behavioral issues and is the age at which veterinarians are trained and are therefore most experienced and comfortable (1, 6). However, studies investigating the safety of EAD anesthetics have reported no significant difference in mortality or morbidity compared with traditional desexing (8) and studies into the risks of long term medical or behavioral issues are conflicting, with some showing a reduction in risk (7, 9, 10) and others showing an increased risk (7, 11, 12).

Medical concerns around PD include the risks presented by pediatric surgery and anesthesia, such as the increased risk of a patient experiencing hypoglycemia and hypothermia (13). Additionally, desexing is known to delay growth plate closure in a range of species (14). However, studies are conflicting when it comes to the relative risk of musculoskeletal disorders associated with PD of cats and dogs and traditional age desexing (9, 14, 15). The risk of obesity is likely increased for cats and dogs that are desexed, however no increase in risk has been found between PD and traditional desexed animals (16). Urinary problems such as urethral obstruction of cats and urinary incontinence in female dogs have also been linked to desexing, with PD suggested as a contributing factor to urinary incontinence in dogs (7, 17). There are also behavioral concerns around PD, with some studies finding noise phobias and sexual behaviors were increased in dogs desexed early (7). However, there have been positive behavioral findings for PD as well, with male cats found to display less aggression and urine spraying (18).

Early age desexing and PD are currently practiced in Australia by animal shelters such as the Royal Society for the Prevention of Cruelty to Animals (RSPCA) (19) and by private veterinarians who service shelters, rescue groups and breeders (1). Prepubertal desexing is not routinely practiced by private veterinary practitioners, however the Australian Veterinary Association's (AVA) policy on desexing does support practitioners who choose to use the procedure (4).

In most states and territories of Australia, the requirement for owners to get their companion animal desexed is not mandatory, but is encouraged through reduced council registration rates. There are, however, several jurisdictions which have introduced mandatory desexing in recent years (Table 1). This is significant for veterinarians, as only 18% of vets in a national survey conducted in 2013 reported being willing to desex dogs and cats at a younger age if government regulations required it (6).

**Table 1:** Details of mandatory desexing requirements in each state and territory of Australia.

**State**	**Is there legislation mandating desexing?**
Australian Capital Territory	Yes Section 74 of the *Domestic Animals Act 2000* makes it an offense to own an undesexed dog over 6 months old or an undesexed cat over 3 months old without a permit
New South Wales	No
Northern Territory	No
Queensland	No
South Australia	Yes Section 42E of the *Dog and Cat Management Act 1995* makes it an offense to own an undesexed dog or cat over 6 months old, with limited exemptions
Tasmania	Yes (cats only) Section 14 of the *Cat Management Act 2009* requires all cats over 6 months of age to be desexed, with limited exemptions
Victoria	No
Western Australia	Yes (cats only) Section 18 of the *Cat Act 2011* requires all cats over 6 months of age to be desexed, with limited exemptions

Section 74 of the Australian Capital Territory's (ACT) *Domestic Animals Act 2000* (the Act) has made PD a legal requirement since 2001, with dogs required to be desexed by 6 months of age. Cats were also originally required to be desexed by 6 months of age, however an amendment to the Act in 2007 saw the legislation change to require all cats to be desexed by 3 months of age. The requirement to desex cats by 3 months is unique in Australia. As mandatory desexing has now been in place in the ACT for over 18 years, we thought it appropriate timing to determine (a) if ACT residents were complying with this legislation, (b) if veterinarians in the ACT were offering this service, and (c) the attitudes and beliefs of veterinarians in the ACT toward prepubertal and mandatory desexing.

## Materials and Methods

A cross-sectional study design was used to survey veterinarians registered in the ACT about their attitudes and behaviors toward prepubertal and mandatory desexing of dogs and cats. The survey was hosted on REDCap, courtesy of The University of Sydney. A link to the survey was emailed to all registered veterinarians in the ACT by the ACT Veterinary Surgeons Board (VSB) and to all members of the AVA ACT Division in September 2017. The survey was open for 1 month.

The questionnaire consisted of 23 questions including six demographic questions, seven cat specific questions, and six dog specific questions (Table 2). Questions were inspired by previous studies (6, 20) with answers structured in a 5-point Likert style. The survey was trialed on three private practice veterinarians and reviewed by the AVA ACT Division committee prior to release. This study was approved by The University of Sydney Human Research Ethics Committee (no. 2017/657) and consent was obtained by implied informed consent via submission of the completed survey.

**Table 2:** Survey of ACT registered clinical veterinarians on their attitudes and behaviors to prepubertal and mandatory desexing.

1. What is your position within the practice? *Associate veterinarian/Practice owner/partner*
2. How many veterinarians (full time equivalent) work at your practice?
3. What age bracket do you fall into? *18–24/25–34/35–44/45–54/55–64/65 or older*
4. How long have you been a practicing veterinarian? *Less than 3 years/3–5 years/5–10 years/10–20 years/More than 20 years*
5. What is the primary focus of your practice? *Small/mixed/equine/exotic/specialist other*
6. How would you describe the area where your practice is located? *Urban/suburban/rural*
7. At what age do you generally advise clients to have their CAT desexed? *Less than 3 months/less than 4 months/less than 5 months/less than 6 months/older than 6 months*
8. How often do you perform CAT desexing prior to 3 months of age? *Always/very often/sometimes/almost never/never/I don't perform desexing procedures*
9. How often do you perform CAT desexing prior to 6 months of age? *Always/very often/sometimes/almost never/never/I don't perform desexing procedures*
10. What are your main concerns regarding prepubertal desexing (less than 4 months) of CATS? Tick all that apply. *Increased anesthetic risks/increased difficulty of surgery/long term behavioral issues/long term medical issues/no concerns/other*
11. Do you currently recommend prepubertal desexing (less than 4 months) to new CAT owners, assuming there are no contraindications to surgery? *Always/very often/sometimes/almost never/never*
12. If you recommend prepubertal desexing (less than 4 months) to some CAT owners, but not all, generally under which circumstances DO you recommend it? Tick all that apply. *If the cat is from a rescue, pet shop or is to be re-homed early/if unwanted pregnancy or early oestrus is probably/if the desexing will prevent unwanted behavior (e.g., spraying)/if the cat will be exposed to FIV or other infectious diseases (e.g., outdoor access)/other/I always recommend prepubertal desexing of cats/I never recommend prepubertal desexing of cats*
13. Do you believe prepubertal desexing (less than 4 months) is an appropriate management strategy to prevent overpopulation of CATS? *Definitely/maybe/neutral/maybe not/definitely not*
14. At what age do you generally advise clients to have their DOG desexed? *Less than 3 months/less than 4 months/less than 5 months/less than 6 months/older than 6 months*
15. How often do you perform DOG desexing prior to 6 months of age? *Always/very often/sometimes/almost never/never/I don't perform desexing procedures*
16. What are your main concerns regarding prepubertal desexing (less than 6 months) of DOGS? Tick all that apply. *Increased anesthetic risk/increased difficulty of surgery/long term behavioral issues/long term medical issues/no concerns/other*
17. Do you currently recommend prepubertal desexing (less than 6 months) to new DOG owners, assuming there are no contraindications to surgery? *Always/very often/sometimes/almost never/never*
18. If you recommend prepubertal desexing (less than 6 months) to some DOG owners, but not all, generally under which circumstances DO you recommend it? Tick all that apply. *If the dog is from a rescue, pet shop or is to be re-homed early/if unwanted pregnancy or oestrus is probable/if the desexing will prevent unwanted behavior (e.g., roaming)/other/I always recommend prepubertal desexing of dogs/I never recommend prepubertal desexing of dogs*
19. Do you believe prepubertal desexing (less than 6 months) is an appropriate management strategy to prevent overpopulation of DOGS? *Definitely/maybe/neutral/maybe not/definitely not*
20. Prior to completing this survey today, were you aware that the ACT had mandatory desexing of cats from 3 months of age and dogs from 6 months of age? *Yes/No*
21. Do you believe prepubertal desexing should be mandatory? *Definitely/maybe/neutral/maybe not/definitely not*
22. Would you be interested in participating in a forum exploring the issues around prepubertal and mandatory desexing in the ACT? *Definitely/maybe/neutral/maybe not/definitely not*
23. Do you have any further comments?

Data from RSPCA ACT was obtained via the software program ShelterBuddy^®^ (RSPCA QLD 2012) by extracting data on all adult cats and dogs admitted to the RSPCA ACT Weston shelter from February 2017 to December 2017. Data from RSPCA NSW was obtained via their annual statistics report submitted to RSPCA Australia for the period of July 2016–June 2017. Z-scores were calculated for RSPCA ACT and RSPCA NSW data, with significant set at *p* < 0.05.

Data from Transport Canberra and City Services (TCCS) was obtained via requests for information between February 2018 and May 2018. TCCS is the ACT government department responsible for domestic animal management, including enforcement of PD legislation.

Survey responses were exported from REDCap to a spreadsheet (Microsoft Excel^®^ 2010) and analyzed using R^®^ (R Foundation for Statistical Computing, Vienna, Austria, 2016). Chi-squared testing was used to assess the relationship between veterinarian age and likelihood of engaging with PD. Ordinal logistic regression testing was used to consider the relationship between demographics and recommended age of desexing. McNemar's test for significance was used to measure the difference between veterinarian's concerns regarding PD for cats vs. dogs. Goodman-Kruskall gamma was calculated to measure the association between respondents reported performance of PD in cats and dogs, Gamma is a measure of concordance suitable to a doubly-ordered contingency table and has a range between −1 and 1. Similarly, the association between scores for other questions were assessed using Kendall's Tau, a rank correlation coefficient also suited to doubly-ordered contingency tables. Tau-b corrects for the presence of ties and also has a range between −1 and 1.

## Results

The survey was emailed to all registered veterinarians in the ACT, which as of July 2017 was 350 veterinarians. Excluding all veterinarians who listed their business address as either a government department, non-practicing, non-veterinary business or NGO, or a non-domestic animal veterinary business, a maximum of 211 of these veterinarians were assumed to be in clinical practice. A total of 59 email recipients activated the survey with 14.9% (52/350) of registered veterinarians completing the survey. Eight-seven percent of respondents identified as small animal practitioners (45/52) in suburban or urban locations (96%; 50/52), representing 25% (52/211) of clinical veterinarians in the ACT. Respondent demographics are summarized in Table 3.

**Table 3:** Demographics of survey respondents.

**Parameter**	**Number of respondents**	**(%)**
**AGE**		
25–34	17	33
35–44	14	27
45–54	12	23
55–64	7	13
65+	2	4
**LENGTH OF TIME AS A PRACTICING VETERINARIAN**		
<3 years	5	10
3–5 years	5	10
5–10 years	9	17
10–20 years	14	27
>20 years	19	37
**POSITION WITHIN PRACTICE**		
Associate	32	62
Practice owner/partner	20	38
**PRIMARY FOCUS OF PRACTICE**		
Small	45	87
Mixed	3	6
Specialist	3	6
Other	1	2
Equine	0	0
Exotic	0	0
**PRACTICE LOCATION**		
Rural	2	4
Suburban	30	58
Urban	20	38
**FTE VETERINARIANS IN PRACTICE**		
0–5	28	54
5–10	21	40
>10	3	6

### Cats

Only 10% (5/52) of respondents recommended that clients desex their cats before 3 months of age. Most respondents advised clients to desex their cat before 5 months of age (38%; 20/52) or 6 months of age or older (40%; 21/52). When asked how often they desexed cats before 3 months of age, 37% stated almost never (19/52) and 13% never (7/52). No veterinarians indicated they always desexed cats before 3 months of age. Respondents were asked if they currently recommended PD to new cat owners, assuming there were no contraindications to surgery. Only 15% (8/52) always recommended PD for cats, with 46% indicating they almost never (33%; 17/52) or never (13%; 7/52) recommended the procedure. Where veterinarians recommended PD to some (but not all) cat owners, many cited population reasons for giving this advice, including if the cat was from a rescue, pet shop or was to be re-homed early (40%; 21/52) or if unwanted pregnancy or early estrus was probable (42%; 22/52). Anesthetic risk in PD was considered significantly more of a concern for veterinarians in cats compared to dogs (*p* = 0.013). Although many veterinarians did not recommend or practice PD, when asked if it was an appropriate management strategy to prevent overpopulation of cats, 77% answered positively with either maybe (37%; 19/52) or definitely (40%; 21/52).

Location of practice influenced a veterinarian's likelihood of recommending older ages of desexing. Veterinarians working in urban practices were less likely to recommend desexing at younger ages compared to veterinarians in suburban or rural practices (Odds Ratio [OR] = 8.99; confidence interval [CI] 2.38–40.66). Veterinarian age was a significant factor (*p* = 0.025) in how often veterinarians performed desexing on cats prior to 3 months of age, although no odds ratio for any particular group reached statistical significance and there was no significant age effect for recommending desexing at this age. Indeed, the relationship between age and frequency of recommending or performing desexing prior to 3 months was complex. Compared to the youngest group of veterinarians (those 25–34), those aged 35–44 and those over 55 tended to both recommend and perform desexing of cats prior to 3 months more often, but those 45–54, somewhat less often. Respondents who indicated they were practice owners or partners were more likely to perform desexing on cats under 6 months of age than associate veterinarians (OR = 7.10; CI = 1.50–37.82). No other significant demographic differences were found.

### Dogs

No respondent indicated they advised clients to desex their dogs before 4 months of age. The majority (71%; 37/52) recommended clients desex their dogs at 6 months of age or older. As with cats, no respondents always desexed dogs before 6 months of age, however 27% (14/52) stated they very often desexed dogs before this age and another 40% (21/52) stated they sometimes did. Respondents were asked if they currently recommended PD to new dog owners, assuming there were no contraindications to surgery. Only 13% (7/52) indicated that they always recommended PD for dogs, with 42% responding they almost never (33%; 17/52) or never (10%; 5/52) recommended the procedure. Of the vets who did recommend PD of dogs to some owners, as with cats, many cited population reasons including if the dog was from a rescue, pet shop or was to be rehomed early (38%; 20/52) or if unwanted pregnancy or early estrus was probable (38%; 20/52). In addition, a further 33% (17/52) indicated they would recommend PD if the desexing would prevent unwanted behavior in dogs, such as roaming. Long-term medical issues in PD were significantly more of a concern for veterinarians for dogs compared to cats (*p* < 0.001).

Respondents were split on whether PD was an appropriate management strategy for dogs, with 33% (17/52) answering definitely and 33% (17/52) choosing to remain neutral on the question. Veterinarian age was a significant factor (*p* = 0.027) in how often veterinarians desexed cats prior to 3 months of age, although no odds ratio for any particular group reached statistical significance. All older age groups recommended desexing at 4–5 months of age more often than the youngest group of veterinarians (aged 25–34); this effect was least pronounced for those aged 45–54. No other significant demographic differences were found.

### Mandatory Desexing

Veterinarians were asked what their main concerns were regarding PD (Table 4). They were also asked if they believed PD should be mandatory in the ACT. Almost half (46%; 24/52) responded definitely not, and 21% (11/52) maybe. Only 12% (6/52) said they definitely thought it should be mandatory. Finally, veterinarians were queried on their knowledge of the mandatory PD desexing laws present in the ACT. The majority of respondents (65%; 34/52) indicated they were aware of the laws, with the remainder (35%; 18/52) saying they were unaware.

**Table 4:** Concerns around prepubertal desexing in dogs and cats in the ACT.

	**Increased anesthetic risk**	**Increased difficulty of surgery**	**Long term behavioral issues**	**Long term medical issues**	**No concerns**	**Other**
Cats	25 (48%)	6 (12%)	6 (12%)	15 (29%)	15 (29%)	6 (12%)
Dogs	15 (29%)	2 (4%)	8 (15%)	32 (62%)	12 (23%)	6 (12%)

There was a moderately strong, significant and positive association between performing PD in cats and performing PD in dogs [Goodman-Kruskall Gamma = 0.53 (0.33–0.74)]. There was a significant, weakly positive association between belief in desexing prior to 4 months of age as an appropriate cat overpopulation management strategy and the frequency of performing (*P* = 0.002; Kendall's Tau = 0.38) and recommending (*P* = 0.003; Kendall's Tau = 0.35) desexing prior to 3 months of age in cats. Likewise, there was a significant, weakly positive association between belief in desexing prior to 6 months as an appropriate dog overpopulation management strategy and the frequency of performing (*P* = 0.021; Kendall's Tau = 0.29) and recommending (*P* = 0.030; Kendall's Tau = 0.25) desexing prior to 6 months of age.

Data on incoming animals was obtained from RSPCA ACT's Weston shelter for a period of 12 months from January to December 2017. The Weston shelter is the only facility in the ACT which accepts stray cats, and one of only two facilities which accepts stray dogs, the other being the Domestic Animal Services pound facility. Adult animal (defined as over 9 months of age) reproductive status (desexed or entire) was assessed as part of the normal veterinary health check on intake. The data revealed on average, only 47.4% (278/587) adult dogs and 41.2% (316/767) adult cats were desexed upon intake to the shelter. RSPCA New South Wales (NSW) reported over the same period that 40.9% (3823/9337) of adult dogs and 38.7% (2468/6378) of cats that entered their facilities were desexed. Dogs entering the RSPCA ACT shelter were significantly more likely (*z* = 3.061; *p* = 0.002) to be desexed than those entering RSPCA NSW facilities. There was no significant difference (*z* = 1.343; *p* = 0.180) between RSPCA ACT and RSPCA NSW with cats entering facilities desexed.

TCCS data on infringements recorded against Section 74 of the Act revealed minimal breaches of the legislation. Unfortunately the data captured in the 16 years from 2001 to 2017 were paper based and unavailable for analysis. Electronic infringement management software was installed in September 2017, with 15 infringements issued under Section 74 (1) for keeping entire dogs without a permit from October 2017 to May 2018. During the same period, RSPCA ACT took in 166 entire dogs. No data were available for cats from TCCS.

## Discussion

Although surveys have previously been conducted into veterinary attitudes and behaviors toward PD in Australia (6, 20), this is the first research conducted specifically into veterinarians operating in a jurisdiction with mandatory PD. The ACT was chosen as a sample population to examine the dynamics between mandatory PD and veterinary attitudes and behaviors. Combined with data on rates of desexed animals and infringements given for breaching the legislation, this research explores the policy space where mandatory PD sits. The *Domestic Animals Act 2000* has required all dogs and cats to be desexed by 6 months since 2001, unless the owner has an exclusion permit. All cats have been required to be desexed by 3 months since the legislation was amended in 2007. Much commentary has taken place in the intervening years, with a review by the AVA in 2007 reporting rates of undesexed animals had not changed. This review concluded mandatory PD as a policy was ineffective, however it did not investigate the causes of this ineffectiveness further (21).

We sought to determine three outcomes from this research. First, we wanted to know if ACT residents were complying with mandatory PD desexing legislation. Second, whether veterinarians in the ACT were offering this service. And third, if the attitudes and beliefs held by veterinarians in the ACT potentially impacted on PD delivery.

The first aim was investigated by interrogating data from RSPCA ACT's Weston shelter. The data revealed that in 2017, on average, only 47.4% of adult dogs and 41.2% of adult cats were desexed upon intake to the shelter. This is despite the introduction of mandatory desexing in the ACT 18 years ago. This period is longer than the average lifespan of both cats and dogs, which means that all dogs and cats should be desexed prior to entry to the Weston facility, if the population was compliant. It is useful to compare these figures with a jurisdiction that does not have mandatory desexing. RSPCA NSW reported in the 2017 calendar year, only 40.9% of adult dogs and 38.7% of adult cats entered their facilities desexed. Although the ACT had significantly more desexed dogs entering RSPCA facilities than NSW, it was still < half of all dogs presented. The rates of desexed cats entering RSPCA NSW facilities were comparable to the rates seen at RSPCA ACT. The demographics of animals entering RSPCA shelters are likely to differ from the general pet population somewhat, however these figures reveal a likely lack of compliance with desexing legislation by pet owners in the ACT.

Data from TCCS for the 7 months from October 2017 to May 2018 showed a low rate of infringements recorded against Section 74 of the *Domestic Animals Act 2000* when compared with the rates of non-desexed dogs entering the RSPCA ACT shelter. Indeed, the 15 infringements reported for those 7 months represent <10% (15/166) of entire dogs which entered RSPCA ACT's shelter during that period, suggesting that enforcement of the desexing legislation in the ACT is minimal.

Our research suggests that the ACT community is failing to comply with the mandatory PD legislation and the government is failing to adequately enforce it. Our next aim was to determine whether veterinarians in the ACT were offering PD and if their attitudes and beliefs impacted PD delivery to the community. The results indicate a veterinary population at odds with the mandatory PD legislation.

Ninety percent of surveyed veterinarians did not recommend cats be desexed before 3 months of age as stipulated in the Act. Half of all veterinarians almost never or never desexed cats before 3 months of age, and no veterinarian always desexed cats before 3 months of age. This may be due to owners not presenting their cats to veterinarians before 3 months of age, or veterinarians not recommending desexing at this age, or a combination of both. Certainly the survey results indicated most vets were not recommending PD in cats, whether this was at 3 months of age as required by the Act or by 4 months of age. This is at odds with veterinarians' views on PD as a management strategy to prevent cat overpopulation, with 40% believing it is definitely a good strategy. It seems as though surveyed vets were aware of the policy reasons for implementing PD but were not applying the policy to their own practices.

Most veterinarians did not recommend PD for dogs. Those veterinarians which did perform PD in dogs were found to also perform PD in cats. This indicates that those veterinarians who practiced PD did so consistently across species, even if they had concerns around PD. Less than one in five veterinarians always recommended PD to new dog owners. One in three stated they almost never recommended the procedure. These results are similar to those found with cats, however the response to PD in dogs was slightly more tempered, potentially because the PD age of 6 months in dogs sits within the traditional desexing range for many veterinarians. Concerns about PD differed between species, with veterinarians more concerned about anesthetic risk in cats compared to dogs. This may be due to a lack of specific training in PD as most veterinarians would have been taught to desex cats at the traditional age of 6 months (22). Unfamiliarity with a procedure promotes caution, however this appears to be unfounded. A large-scale review of mortality rates in EAD showed they were no higher than traditional age desexing (8).

Our research found our sample population of veterinarians in the ACT were not supportive of mandatory desexing. Just under half of all respondents indicated they thought desexing should “definitely not” be mandatory while only 12% thought it “definitely should” be mandatory. As veterinarians are major stakeholders in any law requiring a veterinary procedure, their engagement is essential. Previous research has identified that a legal requirement for PD in a jurisdiction would only cause 18% of veterinarians to desex earlier than they currently recommended (6). This has major implications for jurisdictions like the ACT where PD is mandated. It is difficult for the public to comply with the law if veterinarians choose not to offer the procedure.

Additionally, more than a third of respondents were unaware of the fact that PD desexing was mandatory in the ACT. It seems engagement with veterinarians on animal management legislation in the ACT is poor. Indeed, when veterinarians first register with the ACT VSB, they receive no information about mandatory desexing and microchipping laws in the ACT from the VSB or government.

There are several limitations to this study which may have had an impact on its conclusions. There is always the possibility of non-response bias in anonymous online surveys, with the results only identifying the thoughts of those who took an interest in the issue. Additionally, the response rate to this survey was low, although when taking into account the to the number of non-clinical veterinarians registered in the ACT, the response rate for clinical veterinarians is closer to 24.6% (52/211) than the overall reported rate of 14.9%.

Although the questions were modeled on previous research, some of the answer categories could have had more than one interpretation (e.g., <5 months could also be taken to be <4 months). As with all cross-sectional studies, any associations determined by the study are unlikely to demonstrate a true cause and effect relationship.

Policy developed without adequate stakeholder engagement will struggle to achieve its desired outcomes. In the case of mandatory PD in the ACT, it seems a combination of poor enforcement, stakeholder disengagement and a lack of community awareness has resulted in an ineffective law. Low levels of support from veterinarians for mandatory PD may also restrict the community's access to this procedure. This research suggests engagement and support from veterinarians is critical for mandatory PD to be an effective policy.

## Ethics Statement

This study was approved by The University of Sydney Human Research Ethics Committee (no. 2017/657).

## Author Contributions

BO and BJ: conceptualization, writing, review, and editing. BO: survey development, management, data curation, and project administration.

### Conflict of Interest Statement

BO is employed on a casual basis by RSPCA ACT and also a member of AVA ACT and is registered with the ACT VSB. BJ is employed by RSPCA Australia.

## Abbreviations

ACT: Australian Capital Territory
AVA: Australian Veterinary Association
EAD: Early-Age Desexing
PD: Prepubertal Desexing
RSPCA: Royal Society for the Prevention of Cruelty to Animals.
